# Hyaluronic acid inhibition by 4-methylumbelliferone reduces the expression of cancer stem cells markers during hepatocarcinogenesis

**DOI:** 10.1038/s41598-019-40436-6

**Published:** 2019-03-11

**Authors:** Caecilia H. C. Sukowati, Beatrice Anfuso, Esteban Fiore, Susan I. Ie, Alan Raseni, Fulvia Vascotto, Claudio Avellini, Guillermo Mazzolini, Claudio Tiribelli

**Affiliations:** 1 Fondazione Italiana Fegato, AREA Science Park Basovizza, SS14 km 163.5, 34149 Trieste, Italy; 2 Department of Medicine, University of Udine, Piazzale M. Kolbe 1, 33100 Udine, Italy; 3 Gene Therapy Laboratory, Facultad de Ciencias Biomédicas, Universidad Austral, Avenida Presidente Perón 1500, B1629ODT Derqui-Pilar Buenos Aires, Argentina; 4 Laboratory of Hepatitis and Emerging Diseases, Eijkman Institute for Molecular Biology, Jl. Diponegoro 69, 10430 Jakarta, Indonesia; 5 Institute for Maternal and Child Health - Institute for Research and Health Care Burlo Garofolo, Via dell’Istria, 65, 34137 Trieste, Italy; 6 Department of Medical and Biological Sciences, University Hospital Santa Maria della Misericordia, Piazzale Santa Maria della Misericordia 15, 33100 Udine, Italy

## Abstract

Hyaluronic acid (HA) is a glycosaminoglycan of extracellular matrix related to cell surface which interacts with various cell types. To understand the role of HA during hepatocarcinogenesis, we assessed the effect of the inhibition of HA deposition and its association with heterogeneous hepatocellular carcinoma (HCC) cells. In this study, we used transgenic mice C57BL/6J-Tg(Alb1HBV)44Bri/J (HBV-TG) and normal C57BL/6 J (WT) for *in vivo* study, while HCC cells Huh7 and JHH6 as *in vitro* models. Both models were treated with an HA inhibitor 4-methylumbelliferone (4MU). We observed that 4MU treatments in animal model down-regulated the mRNA expressions of HA-related genes Has3 and Hyal2 only in HBV-TG but not in normal WT. As observed *in vivo*, in HCC cell lines, the HAS2 mRNA expression was down-regulated in Huh7 while HAS3 in JHH6, both with or without the presence of extrinsic HA. Interestingly, in both models, the expressions of various cancer stem cells (CD44, CD90, CD133, and EpCAM) were also decreased. Further, histological analysis showed that 4MU treatment with dose 25 mg/kg/day reduced fibrosis, inflammation, and steatosis *in vivo*, in addition to be pro-apoptotic. We concluded that the inhibition of HA reduced the expressions of HA-related genes and stem cells markers in both models, indicating a possible modulation of cells-to-cells and cells-to-matrix interaction.

## Introduction

Hepatocellular carcinoma (HCC) is a major health problem, being the second most common cause of cancer-related death worldwide^1^. More than 50% HCC cases are related to chronic hepatitis B virus (HBV) infection and chronic HBV carriers have a 100-fold relative risk for developing HCC, with an annual incidence rate of 2–6% in cirrhotic patients^2^.

One of the main characteristics during hepatocarcinogenesis is the disturbance in the liver architecture, shown by the accumulation of extracellular matrix (ECM) in the liver parenchyma. Hyaluronic acid (hyaluronan, HA) is a linear, large and ubiquitous non-sulfated glycosaminoglycan of the ECM^3^. HA is found in almost all tissues but it is especially increased in those with active cell proliferation, regeneration, and repair, such as embryonic, inflamed, and tumor stroma tissues^4,5^. It is one of the markers of fibrosis in liver diseases^6,7^ and high preoperative serum HA levels can be used to predict poor prognosis in patients with HCC after hepatic resection^8^.

Previously published studies in various diseases models cancers had shown the relevance of HA blocking by 4-methylumbelliferone (4MU). 4MU is a derivate of 7-hydroxycoumarin (umbelliferones), substances present in many species of plants, especially umbelliferae, fabaceae, and oleaceae. It is dietary supplement since the 1990s to support liver function and detoxification, as well as in clinical trials in patients with chronic hepatitis B and C.

It had been demonstrated that a 12-weeks 4MU supplementation was also shown to ameliorate hypertriglyceridemia and hyperglycemia in mice receiving high-fat diet^9^. A previous study had shown that treatment of HCC cells with 4MU significantly reduced tumor cell proliferation and induced apoptosis, while primary cultured hepatocytes remained unaffected. In addition, 4MU therapy reduced hepatic and systemic levels of HA. Most important, tumors systemically treated with 4MU show extensive areas of necrosis, inflammatory infiltrates and a 2–3-fold reduction in the number of tumor satellites^10^.

HA links to cellular receptor CD44 and their interaction in the high-fat diet in mice promoted cancer progression and therapy resistance in different types of cancer, including in HCC^11,12^. CD44, an adhesion molecule, is a multifunctional cell-surface glycoprotein involved in cell-cell interactions, cell adhesion, and cells migration. It interacts with different cells populations and it is one of the phenotypical markers of cancer stem cells (CSC), either alone or in combination with other CSC markers. However, little is known about the inhibition of HA, CD44, and other cells populations during hepatocarcinogenesis. In this report, we investigate the effect of the inhibition of HA retention in the progression of HCC and its direct/indirect correlation with heterogeneous CSC populations.

## Results

### Down-regulation of HA genes by 4MU in HBV-TG mice

HA deposition was evident in the liver of HBV-transgenic mice (HBV-TG), especially in the hepatic periportal area while this accumulation was not noticed in wild-type mice (WT).

**Figure 1:**
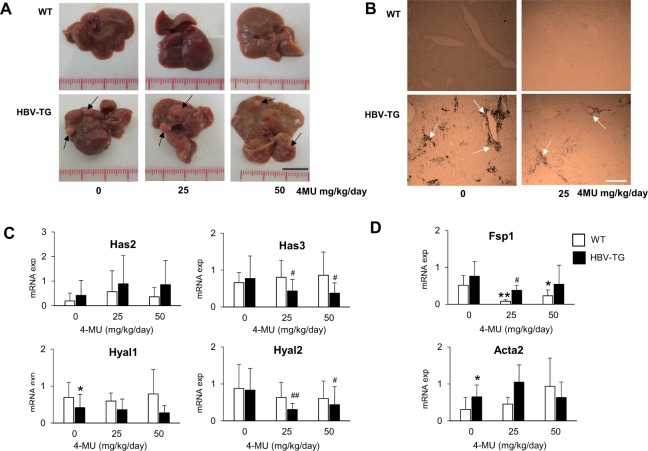
Effect of 4MU treatment in HBV-TG mice. (**A**) Macroscopic appearance of liver after 12 weeks treatment of 25 and 50 mg/kg/day 4MU (scale bar = 1 cm). (**B**) HA staining of the liver before and after treatment. Magnification: 200X (scale bar = 300 µm). (**C**) Relative mRNA expressions of hyaluronan synthases Has2 and Has3, and hyaluronidases Hyal1 and Hyal2. (**D**) Relative mRNA expressions of fibrosis and ECM genes Fsp1 and Acta2. Each mRNA expression of target genes was normalized to reference genes Gapdh and Actb. Statistical analysis: *p < 0.05, **p < 0.01 compared to WT 0 mg/kg/day, ^#^p < 0.05, ^##^p < 0.01 compared to TG 0 mg/kg/day. WT = wild type mice, HBV-TG = HBV-transgenic mice.

After 4MU treatment with 25 mg/kg/day (0.02%) and 50 mg/kg/day (0.04%) for 12 weeks, we still observed hepatic nodules in HBV-TG. 4MU showed a mild inhibitory effect on the growth of the tumor. Due to the fluorescence effect of 4MU, a yellowish color of the liver was noticed in all treated animals (Fig. 1A). As shown in Fig. 1B, the HA was still noticed in the liver of treated animals. No animals showed any adverse reactions during treatment; only in a group of 50 mg/kg/day HBV-TG mice, a slight increase (10%) of body weight was observed (Table 1).

**Table 1:** Serum markers and body weight in animal model after 4MU treatment.

mg/kg/day	WT			HBV-TG		
	0	25	50	0	25	50
ALT (IU/L)	47 ± 20	73 ± 81	110 ± 136	186 ± 145	246 ± 176	314 ± 192
AST (IU/L)	131 ± 79	123 ± 40	141 ± 66	233 ± 133	270 ± 163	312 ± 177
LDH (IU/L)	925 ± 243	2002 ± 480**	2129 ± 638***	1453 ± 339	2083 ± 440**	2284 ± 992*
Weight (g)	40 ± 6	40 ± 4	40 ± 5	39 ± 5	37 ± 3	43 ± 8

Statistical student’s *t* test: *p < 0.05, **p < 0.01, ***p < 0.001 compared to CTRL 0 mg/kg/day of each strain.

The HA-related genes were analyzed in 133 tissue samples (WT n: 20, 23, 21 and HBV-TG n: 23, 26, 20 samples, for treatment 0, 25, and 50 mg/kg/day, respectively). At basal level, HBV-TG mice had higher mRNA expression of HA synthases Has2, and lower hyaluronidase Hyal1 (p < 0.05), as compared to WT. After treatment, RTqPCR data showed that the mRNA expressions of Has3, Hyal1, and Hyal2 were decreased only in HBV-TG by around 35%, 50%, and 65%, respectively. 4MU treatment did not result in any significant effects to the Has3, Hyal1, and Hyal2 of the WT animals. However, in contrast, Has2 mRNA was up-regulated in both strains with high variability (Fig. 1C).

Further analysis of the ECM genes showed that 4MU treatment also reduced the expressions of Fsp1 (fibroblast specific protein 1) in both WT and HBV-TG mice, with the highest effect in WT (p < 0.01). However, this down-regulation was not noticed for Acta2 (alpha smooth muscle actin) (Fig. 1D).

### Improvement of fibrosis stages

**Figure 2:**
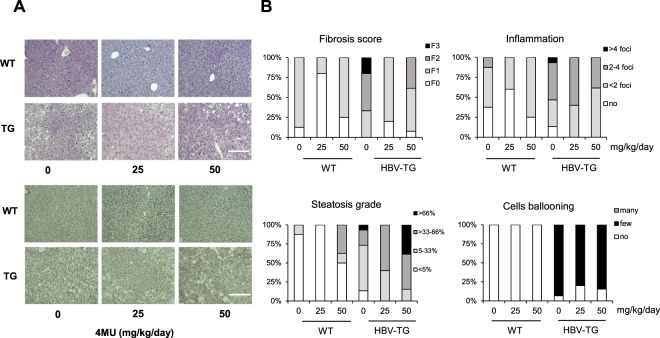
4MU treatment improves histology in HBV-TG mice. (**A**) Representative pictures of H&E staining (upper panel) and reticulum staining (lower panel) (scale bar = 200 µm). (**B**) The percentage of fibrosis score, steatosis grade, and inflammation scores in the liver. WT = wild type mice, HBV-TG = transgenic mice.

Histological analysis was performed in 21 WT (8, 5, and 8 slices for 0, 25, and 50 mg/kg/day, respectively) and 26 HBV-TG (8, 5, and 13 slices for 0, 25, and 50 mg/kg/day, respectively) as shown in Fig. 2A. The histological scoring system was based on a previous study^13^.

As shown in Fig. 2B, at basal level the proportion of HBV-TG mice with fibrosis stages F3, F2, and F1 were 20%, 50%, and 30%, respectively. After treatment with 4MU of 25 mg/kg/day, this proportion was significantly changed as 80% of animals were F1, while F2 and F3 were not noticed. However, 40% F2 was detected in the group treated with highest 4MU concentration (50 mg/kg/day), even though none of the mice had an F3 stage. The similar beneficial effect was noticed in WT, 4MU treatment with 25 mg/kg/day showed a better result than 50 mg/kg/day, decreasing F1 from 90% to 20% and 70%, respectively.

We also observed the favorable effect of the 25 mg/kg/day treatment on both steatosis and inflammation although administration of 50 mg/kg/day increased the percentage of steatotic cells (40% vs. 10% in control group) which might be correlated with the increase of body weight. The percentage of cells ballooning was also slightly decreased (from 90% to 80%).

### Quantification of serum transaminases and LDH

The quantification of serum ALT, AST, and LDH in all 56 animals was shown in Table 1. As we had previously reported^14^, HBV-TG animals had a significantly higher level of serum ALT (186 ± 145 vs 47 ± 20 IU/L), AST (233 ± 133 vs 131 ± 79 IU/L), and LDH (1453 ± 339 vs 925 ± 243 IU/L) compared to WT, indicating a progressive hepatic damage in these mice.

After treatment, we noticed that the level of serum ALT incresead along with the increase of 4MU concentration. This increase was more noticeable, though not statistically significant, in WT than in HBV-TG. The level of AST remained stable while LDH activity in both mouse models progressively increased, reaching for around 2-fold higher in WT (mean values: 925 to 2129 IU/L, p < 0.01) and 1.6-fold higher in HBV-TG (mean values: 1453 to 2284 IU/L, p < 0.05).

### Down-regulation of HA genes by 4MU in HCC cell lines

The relative mRNA expression of HA synthases HAS2 is significantly higher in Huh7 compared to JHH6, (around 700-fold, p < 0.01), while the expression of HAS3 is high in JHH-6 (around 13-fold compared to Huh7). For hyaluronidases, the HYAL1 and HYAL2 mRNA expressions of Huh7 were around 2-fold higher than those of JHH6 (p < 0.05). Both cell lines did not express HAS1 (data not shown).

**Figure 3:**
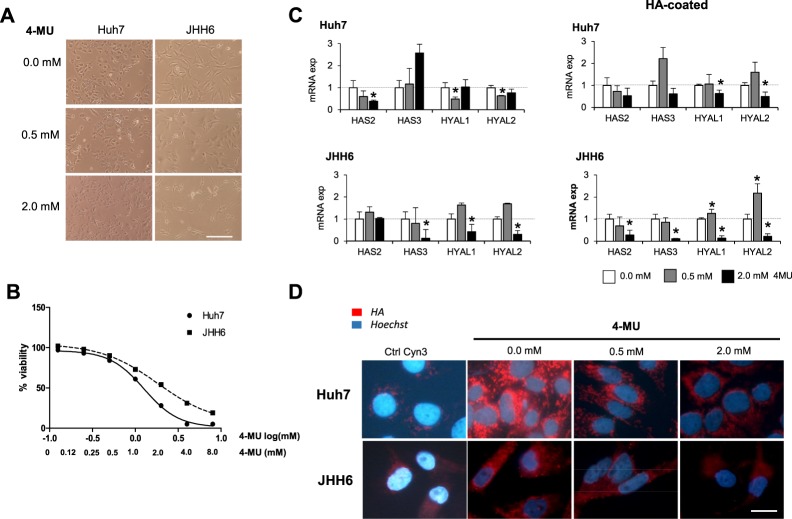
Effect of 4MU treatment in HCC cells JHH6 and Huh7. (**A**) Morphological cellular changes after treatment (scale bar = 200 µm). (**B**) Dose-dependent cytotoxicity of 4MU with concentration ranging from 0.0–8.0 mM by MTT test. Concentration was shown as log[mM]. (**C**) Relative mRNA expressions of hyaluronan synthases HAS2 and HAS3, and hyaluronidases HYAL1 and HYAL2 after treatment of 0.5 and 2.0 mM of 4MU. mRNA expression was normalized to reference genes 18S and ACTB. Statistical analysis: *p < 0.05, **p < 0.01 compared to untreated control of each cell line (0.0 mM = 1.0) (**D**) Presence of cytoplasmic HA (scale bar = 20 µm).

After 4MU treatment for 24 hours, by using MTT test, we noticed that the cytotoxicity of 4MU was dose-dependent, both for Huh7 and JHH6. Cells viability was gradually decreased along with the increased concentration of 4MU. In low concentration 0.5 mM (log[mM] −0.301), both cell lines showed a comparable viability for around 85%. Cells morphology remained similar with control. At high concentration 2 mM (log[mM] 0.301) the JHH6 showed higher viability compared to Huh7, for around 54% and 28% respectively. Furthermore, the cells lost their morphological origins (Fig. 3A and B).

Gene expression analysis showed that in both cell lines, the mRNA expressions of HYAL1 and HYAL2 were decreased at 2 mM 4MU treatment. In Huh7, a significant change was already noticed at low concentration treatment 0.5 mM. For HAS2 and HAS3, the pattern of down-regulation in each cell line was different, it found to be depended on their basal expression. In Huh7 with high HAS2, 4MU treatment down-regulated HAS2 for 60% (p < 0.05), but not for HAS3. In contrary, In JHH6 with high HAS3, 4MU significantly down-regulated HAS3 for around 85% (p < 0.05). The maximum down-regulation for both genes in both cell lines was detected at 2 mM treatment (Fig. 3C).

By using immunofluorescence against the HA-binding protein, we observed positivity of cytoplasmic HA of both cells lines. As shown in Fig. 3D, the 4-MU treatment clearly reduced the accumulation of HA.

Interestingly, when the extrinsic HA was introduced by using a HA-coated plate (8,000–15,000 MW), the expressions of HAS2 and HAS3 in both cells were even reduced after treatment. In JHH6, HAS2 was also significantly reduced. For hyaluronidases, when the HA was present, the 4MU increased the expressions of these enzymes, probably in an effort to destroy the HA (Fig. 3C).

### Decrease of the CSC markers in HBV-TG mice

To explore the significance of the inhibition of HA in the profile of hepatic CSC, we further examined the expressions of CSC markers in both HCC cell lines and animal model.

In the *in vivo* animal model, the CSC markers genes were analyzed in 133 tissue samples (WT n: 20, 23, 21 and HBV-TG n: 23, 26, 20 samples, for treatment 0, 25, and 50 mg/kg/day, respectively). In comparison to WT, the HBV-TG mice showed a significantly higher expressions of Cd44, Cd90, Cd133 (p < 0.05), and Epcam (p < 0.01).

**Figure 4:**
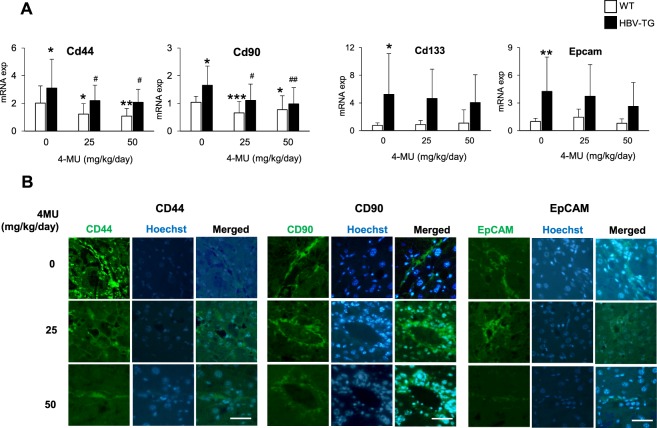
4MU treatment reduces the expressions of CSC markers in mouse model. (**A**) Relative mRNA expressions of CSC markers Cd44, Cd90, Cd133, and Epcam in HBV-TG and WT mice. Statistical analysis: *p < 0.05, **p < 0.01 compared to WT 0 mg/kg/day, ^#^p < 0.05, ^##^p < 0.01 compared to TG 0 mg/kg/day. (**B**) Representative of immunofluorescence images of protein CD44, CD90, and EpCAM in HBV-TG (scale bar = 50 µm). WT = wild type mice, HBV-TG = HBV-transgenic mice.

The hepatic mRNA expression of Cd44 was significantly down-regulated by the 4MU treatment in both WT and HBV-TG mice. The down-regulation of Cd44 was accompanied by the decrease of Cd90 (Fig. 4A).

An interesting behavior was noticed for Cd133 and Epcam as their expressions were decreased only in TG animals, while the treatment had not effect in WT animals (Fig. 4A). Importantly, since we analyzed different portions of hepatic tissues of each TG mouse, we observed that the down-regulation of CSC markers was clearly observed in hepatic nodules compared to non-nodules. Representative images of the positivity of CSC markers CD44, CD90, and EpCAM proteins are shown in Fig. 4B.

### Decrease of the CSC markers in HCC cell lines

**Figure 5:**
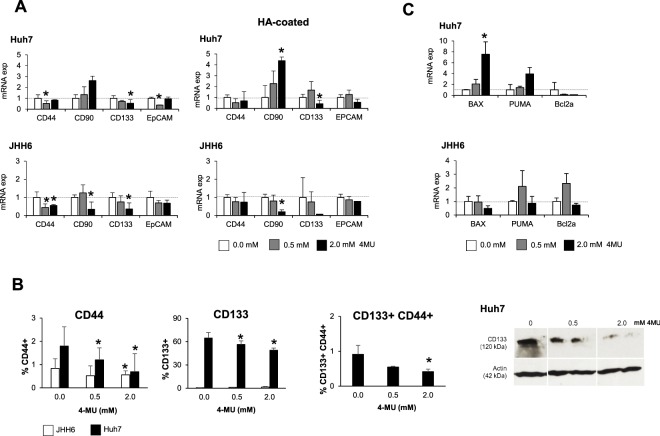
4MU treatment reduces the expressions of CSC markers in HCC cells JHH6 and Huh7. (**A**) Relative mRNA expressions of CSC markers CD44, CD90, CD133, and EpCAM after treatment. (**B**) Flow cytometric analysis on the number of CD44+, CD133+, and CD133+CD44+after treatment (left). Western blot representative of CD133 protein of Huh7 (right). Full-length Western blot is presented in Supplementary Fig. S1. (**C**) Relative mRNA expressions of apoptosis genes after treatment. mRNA expression was normalized to reference genes 18S and ACTB. Statistical analysis: *p < 0.05 vs. untreated control of each cell line (0.0 mM = 1.0).

In HCC cell lines model, the expression of CD44, the receptor of HA, was significantly down-regulated (around 50%) in both cell lines after 0.5 mM 4MU treatment (p < 0.05). Interestingly, the expression of the CD133/Prominin-1, a cell surface marker of hepatic CSC, was also down-regulated (60% and 50% less than control for the JHH6 and Huh7, respectively, p < 0.05) (Fig. 5A).

This mRNA result was confirmed by flow cytometry where we observed that the percentage of CD44+ cells decreased from 0.8% to 0.5% in JHH6 and from 1.8% to 0.7% in Huh7 after treatment (p < 0.05). The reduction of CD133 was also noticed. The percentage of CD133+ in Huh7 was significantly decreased from 65% to 49% (p < 0.05). The reduction of double-positive CD133+ CD44+ was also observed in Huh7, the percentage of this cell population decreased for around 40% and 50% for 0.5 mM and 2.0 mM (p < 0.05). The protein expression of the CD133 was confirmed by Western Blot (Fig. 5B). The mRNA expression of EpCAM was significantly decreased in Huh7 and in lower extent in JHH6; mRNA expression of CD90 was decreased only in JHH6.

Interestingly, in Huh7, the decrease of CD133 and EpCAM after 4MU treatment was also accompanied by the increase of pro-apoptotic genes PUMA and BAX and the decrease of anti-apoptotic gene  Bcl2a (Fig. 5C).

## Discussion

In this study we report the effect of 4MU, an HA inhibitor, in the down-regulation of HA-related genes and the reduction of HA accumulation in two sets of experimental models, an HCC-transgenic mouse model and human HCC cell lines. For the animal model, we employed the HBV-transgenic mouse model C57BL/6J-Tg(Alb1HBV)44Bri/J (HBV-TG). This strain contains the HBV fragment codifies the envelope protein (HBsAg) and its intrahepatic concentration corresponds with the incidence of HCC^15^. Our previous study showed early liver inflammation caused by the HBsAg accumulation together with an increased expression of the markers of hematopoietic stem cells markers in the liver. The progression of liver damage up-regulated different CSC populations, such as EpCAM and CD133^14^.

Regardless of the etiologies, the main risk factor of HCC development is liver cirrhosis. It is preceded by a progressive fibrosis, characterized by the scarring of fibrous connective tissue as a reparative response to injury or damage. The level of HA has been used as a non-invasive biomarker to assess liver fibrosis (reviewed in^16^). In chronic hepatitis B, various studies had demonstrated that HA level increased with the increase of the stages of liver fibrosis, to be highest in liver cirrhosis (F4)^17–23^. It was reported that low serum HA level was associated with spontaneous HBsAg clearance^24^ while in HCC, high serum HA levels (100 ng/ml or above) were associated with shorter recurrence-free survival. Interestingly, also in patients with HCC without severe liver fibrosis, serum HA level was correlated with the presence of multiple tumors^8^. In tissues, by using immunostaining, higher expression of HA was significantly exhibited in HCC tissues compared with paired normal liver tissues. High HA expression was also correlated with poor overall survival, large tumor size, and poor tumor differentiation^12^.

In our animal study, we started the 12 weeks treatment in 9-month old mice, an age when in HBV-TG mice begin the initial development of liver dysplasia leading to HCC at 12-month of age. The 12-month old HBV-TG mice had higher expression of Has2 and lower expression of Hyal1 compared to WT mice. And as expected, HBV-TG mice showed a high incidence of liver fibrosis stages F2 and F3 and the deposition of HA in the liver. They also had higher expression of fibrosis marker Acta2 and Fsp1. After treatment with 4MU we observed the decrease of HA deposition in the liver. Importantly, it was accompanied by a significant reduction in the incidences of F2 and F3 in HBV-TG mice, especially in 25 mg/kg/day treatment. It is also noteworthy to notice that the reversal of hepatic histological states was also observed by the improvement of steatosis and inflammation, as well as cells ballooning.

Our gene expression analysis showed that 4MU treatment reduced the mRNA expressions of HA-synthesis (HAS) Has3 and HA-degradation (hyaluronidase) enzyme Hyal2, while it slightly down-regulated Hyal1. Has3 codifies for the production of low molecular weight HA that has been shown to induce inflammatory cytokines^25^. Our data on the down-regulation of Has3 mRNA and the reduction of fibrosis and inflammation is consistent with previous animal studies in lung and colitis model^25–27^. Previously, it was shown that Has3 mRNA in the liver was increased after carbon tetrachloride exposure for fibrosis induction in normal mice^28^.

One of the important findings was that the down-regulation of HA-related mRNAs were observed only in treated HBV-TG mice, but not in WT mice, suggesting that 4MU does not affect the HA synthesis in normal hepatic cells. This conclusion is in line with previous reports showing the anticancer effect of 4MU in HCC. In xenograft HCC models, 4MU significantly reduced tumor cell proliferation while primary cultured hepatocytes remained unaffected^10^. 4MU also reduced the expression of CD31 and inhibited systemic level of VEGF and IL-6 production was dramatically reduced in isolated Kupffer cells^29^.

To better understand the data obtained *in vivo*, we performed in *vitro* experiments using two human HCC cell lines with different degree of differentiation, Huh7 and JHH6. We observed that HAS2 mRNA was highly expressed in Huh7, a well-differentiated HCC cell line, while on the contrary HAS3 was highly expressed in JHH6, a poor differentiated HCC cell line. As exhibited in mouse models, 4MU treatment reduced the expressions of HAS *in vitro*: it down-regulated the high basal level of HAS2 mRNA in Huh7, and the high level of HAS3 mRNA in JHH6. This finding suggests that the effect of 4MU in targeting HA-enzymes depends on the initial level of HAS in the cells.

As in mouse model, 4MU also down-regulated the mRNA of hyaluronidases HYAL1 and HYAL2 in both cell lines. The results in HCC are in line with previous reports *in vitro* in breast, melanoma, ovarian, squamous carcinoma cell lines^30,31^ and *in vivo* mouse models of bladder, malignant pleural mesothelioma, malignant peripheral nerve sheath, and mostly in pancreatic cancer^32–37^.

To observe the effect of 4MU in the deposition of extracellular HA, we added gene expression analysis of HA-related enzymes in the presence of extrinsic HA. By treating the cells on the HA-coated plate (8,000–15,000 MW), we observed a higher expressions hyaluronidases compared to cells grown in non-coated plates, probably in an effort to destroy the presence of HA. In addition, the reduction of both HAS2 and HAS3 were also noticed in JHH6.

Further, we checked whether the reduction of HA enzymes mRNA was also accompanied by the decrease of its cell receptor CD44. The CD44 molecule (Indian blood group) is a multifunctional cell-surface glycoprotein involved in cell-cell interactions, cell adhesion, and cells migration. It serves as an adhesion/homing protein receptor for the HA in the ECM^38^. The expression of CD44 in HCC is associated with poor prognosis and tumor aggressiveness^39,40^. Our data in human samples showed that CD44 mRNA was increased in HCC as compared to normal liver tissues (data not shown). Further, CD44 has been implicated as one of CSC markers in HCC, as a single marker as well as in combination with other CSC markers.

In bone metastasis cell lines, it had been reported that CD44 cell acts as the CSC and promotes bone metastases by enhancing tumorigenicity, cell motility, and HA production^41^. Accordingly, we also investigated whether other CD44-associated CSC markers, CD90, CD133, and EpCAM^42–45^, were affected by 4MU treatment. We observed that both in mice model and in human HCC cell lines, CD44 mRNA was decreased after 4MU treatment. The decrease of CD44 mRNA in cells Huh7 and JHH6 was confirmed by the reduction of CD44+ cells using flow cytometry where the CD44-positivity was reduced by about 50% after treatment in both cell lines.

Besides the decrease of CD44 cells, we found the 4MU treatment also reduced various HCC CSC markers. In mouse models, 4MU significantly decreased the expression of Cd90 in both HBV-TG and in WT mice in a concentration-dependent manner, as for Cd44. Interestingly, even though we did not reach significant value due to the high heterogeneity of mRNA expressions, we observed the mRNA expressions of Cd133 and Epcam were also down-regulated.

In line with the *in vivo* model, 4MU reduced the expressions of CD133 and EpCAM in both Huh7 and JHH6, while the CD90 mRNA expression was reduced only in JHH6. As the down-regulation of CD133 was found to be the most significant, we performed a quantification of CD133+ cells and CD133 protein expression in Huh7 by using flow cytometry and Western blot, respectively. Both analyses confirmed the down-regulation of CD133 molecule after 4MU treatment.

The mechanisms by which 4MU inhibits the properties of CSC is still unclear. In oral squamous cell carcinoma cell lines, a population identified as rapid adherent cells (RAC) to collagen-IV was enriched for its high tumorigenic ability. Using scanning electron microscopy, 4MU was shown to altered membrane morphology of the RAC and significantly decreased their membrane capacitance^46^.

In the aforementioned bone metastasis study, 4MU decreased CD44 together with CSC tumor sphere and osteoclast-like cell formation *in vitro*^41^. A study in colorectal, osteosarcoma, and lung cancer cell lines showed that irradiation induced the expression of HAS2, resulting in their radioresistance, one of the hallmarks of the CSC^47^. Here, we observed that 4MU increased the pro-apoptosis genes Bax and Puma, while decreased the anti-apoptosis gene Bcl2a in Huh7. It was known that CSC CD133 is correlated with anti-apoptosis property^48,49^. However, the effect of 4MU in the epithelial mesenchymal transition (EMT), another hallmark of CSC, was unclear. Perhaps, a treatment for 24 hours was not sufficient to block the EMT (supplementary material).

Our study in a transgenic model is in line with a recent data on the 4MU treatment in an orthotopic HCC model established in fibrotic livers. 4MU, in combination with an adenovirus encoding interleukin-12 genes elicited a potent antitumor effect accompanied by the reduction of mRNA levels of CSC markers^50^.

In this study, we found that 4MU therapy in this *in vivo* model was acceptable for both experimental doses of 25 and 50 mg/kg/day. Even though we did not see the disappearance of liver nodules, a clear improvement in liver histology and the marked decrease of CSC markers in nodules was observed supporting the anticancer properties of 25 mg/kg/day of 4MU. A 4MU study in osteosarcoma mice model showed only a mild inhibitory effect on the growth of the primary tumor but a marked inhibition (75% reduction) in lung metastasis^51^.

In conclusion, we demonstrated that the reduction of HA by 4MU lowered HA-related genes that reduced deposition of HA in HCC. The inhibition of HA was accompanied by a reduction of CSC markers CD44 and CD133, as well as CD90 and EpCAM cells, indicating a possible mechanism of HA in cells-to-cells and cells-to-matrix interaction.

## Materials and Methods

### Animal model

Fifty-six male Hepatitis B Virus (HBV)-transgenic mouse C57BL/6J-Tg(Alb1HBV)44Bri/J (HBV-TG, n = 28) and its wild-type counterpart C57BL/6 J (WT, n = 28) were maintained in the animal facility of the University of Trieste. Mice were randomly selected for the treatment group. The animal procedure was performed in accordance with the proper care and use of laboratory animals. The study protocol was approved by the Ethical Committee of the University of Trieste and the Ministry of Health of the Republic of Italy (Decreto no: 57/2012 – B).

### HCC cell lines

Human HCC cell lines Huh7 (JCRB0403) and JHH6 (JCRB1030) were obtained from the Japan Health Science Research Resources Bank (HSRRB, Tokyo, Japan). Huh7 cells were grown in DMEM medium (high glucose) supplemented with 10% (v/v) FBS, 1% L-glutamine and 1% antibiotics. JHH6 cells were grown in Williams’ E medium supplemented with 10% (v/v) FBS, 1% L-glutamine and 1% antibiotics. The cultures were maintained at 37 °C in a humidified 5% CO_2_ incubator and when they reached 80–90% confluence they were routinely expanded by 0.05% trypsin detachment.

### Inhibition of HA deposition by 4MU

For *in vivo* study, 9 month old mice, for both HBV-TG (initial development of liver dysplasia) and WT, were given 4MU (Sigma Aldrich, Milan, Italy) with concentration 25 mg/kg/day (0.02%) and 50 mg/kg/day (0.04%) by oral administration in drinking water for 12 weeks. Mice without 4MU administration were used as control group (0 mg/kg/day). At the end of treatment, animals were weighted and euthanized in deep anesthesia. Blood and liver tissues were collected immediately after sacrifice. Three different portions of liver were sliced and collected in sterile tubes for subsequent gene, protein, and histochemical analysis. Body and liver weight were measured.

For *in vitro* study, HCC cell line Huh7 and JHH6 cells were cultured in a 6-well plate with or without 0.2 mg/ml HA (8,000–15,000 MW) (Sigma) with an initial concentration of 100,000 cell/ml and 50,000 cell/ml, respectively, for 24 hours. 4MU was dissolved in stock solution and subsequently in the growth medium. 4MU concentration ranging from 0 to 8 mM was given for treatment for 24 hours. After treatment, the cells were collected for flow cytometry analysis, total RNA extraction, and total protein extraction. The protocol of HA-coating was based on a previous study^52^.

### Quantification of serum transaminases and lactate dehydrogenase

After collection, mice sera were separated from whole blood and they were stored at −80 °C until analyzed. Serum alanine aminotransferase (ALT), aspartate aminotransferase (AST), and lactate dehydrogenase (LDH) were quantified with the standard enzymatic methods (Roche Cobas Analyzer) at the IRCCS Burlo Garofalo children hospital.

### Histology

Liver tissues were fixed in 4% formalin for at least 24 hours. The samples were included in paraffin block with the automated Sakura method at the Santa Maria della Misericordia Hospital. The fixed slices were subjected to hematoxylin & eosin (HE) and reticulum staining to assess the staging of liver fibrosis. Histological analysis was examined by a pathologist. Diagnostic and the scoring system in histological analysis was referred to the validated consensus of non-alcoholic steatohepatitis and fibrosis^13^.

### HA staining

HA staining of mouse liver paraffinated slices was performed as described previously^10^. Briefly, paraffin liver sections were incubated with 3% H2O2–methanol for 30 min at room temperature to block endogenous peroxidase, followed by avidin, biotin and protein-blocking solution (Vector, Buenos Aires, Argentina), and bHA-BP (# 385911, Calbiochem) in BSA–PBS. Negative controls was stained with bHA-BP and pretreated with 100 U/mL of Streptomyces hyaluronidase (Calbiochem). Peroxidase complex (Sigma-Aldrich, Buenos Aires, Argentina) was used as a revealing system.

In human HCC cell lines, the cytoplasmic HA positivity were detected by hyaluronic acid binding protein (HABP). After 4MU treatment, cells grown in cover slip were fixed in 3% paraformaldehyde. Cells were then blocked, permeabilized and quenched by 1% BSA, 0.1% Triton X, 50Mm Glycine in PBS. Incubation with HABP-biotin labeled (5 ug/ml) (Merck, Milan, Italy) that binds specifically and strongly to HA ≥ 2000 MW was done overnight in 4 °C in a humid chamber. The day after, cells were washed and incubated with streptavidin-Cyn3 for 1 hour in room temperature. The nuclei were stained by Hoechst 33258. Controls without HABP (ctrl Cyn3) were included. Images were generated using fluorescence microscope Leica DM2000 (Leica, Wetzlar, Germany).

### Total RNA isolation

Total RNA from HCC cell lines and mouse liver tissues was extracted using the TriReagent (Sigma Aldrich) according to the manufacture’s protocol. RNA was quantified at 260 nm in a DU®730 spectrophotometer (Beckman Coulter, Fullertone, CA, USA). The RNA purity was evaluated according to MIQE guidelines^53^ by measuring the ratio A260/A280 with appropriate purity values between 1.8 and 2.0.

### Reverse Transcription quantitative real time PCR (RT-qPCR)

cDNA synthesis was obtained by reverse transcription (RT) of 1 µg of total RNA using High Capacity cDNA Reverse Transcription Kits (Applied Biosystems, Foster City, CA, USA) according to the manufacture’s suggestions.

RT-qPCR was performed using iQ SYBR Green Supermix protocol (Bio-Rad Laboratories, Milan, Italy). Each PCR amplification was carried out in 15 µL reaction volume containing 25 ng of cDNA, 1x iQ SYBR Green Supermix, and 250 nM gene-specific sense and anti-sense primers. Reactions were run on a Bio-Rad iQ5 real-time PCR detection system (iCycler IQ5 software, version 3.1; Bio-Rad) together with two reference genes. The list of the primers description and sequences were shown in Supplementary Materials.

### Flow cytometry of CSC markers

The presence of CSC surface marker antigens was detected using antibodies CD90/THY1 (Stem Cell Technologies, Vancouver, BC, Canada), CD133/PROM1 (Miltenyi Biotec GmbH, Bergisch Gladbach, Germany), and CD44 (Abcam, Cambridge, UK). Flow cytometric analysis was performed immediately on a FACSCalibur flow cytometer (Becton Dickinson, NJ, USA). A total of 10,000 events were analyzed per sample. Data were presented as a mean for at least three independent experiments.

### Immunofluorescence of CSC markers

For mouse liver tissues, after de-paraffinization with xylene and rehydration with gradual concentration of ethanol, antigen retrieval was performed by microwave heat in sodium citrate pH 6.0. The autofluorescence background of paraffin tissues was quenched by combination of photo bleaching and Sudan Black B dye^54^. Direct immunostaining was performed with fluorochromes-conjugated antibodies against mouse CD44 (Miltenyi Biotec) CD90 (Santa Cruz Biotechnology, Californa, USA) and CD326/EpCAM, Miltenyi Biotec). Proteins positivity was observed by using a fluorescence microscope Leica DM2000.

### Protein extraction and Western Blot

Western Blot was performed for CD133. Total protein of Huh7 was extracted by using cell lysis buffer. Twenty ug of total protein of Huh7 were size-separated, together with molecular weight standards (Fermentas) by (SDS–PAGE) on polyacrylamide gel, using a Mini Protein III Cell (Bio-Rad). After SDS–PAGE, proteins were electro-transferred with a semi-dry blotting system onto immune-blot PVDF membranes (Bio-Rad) using a Mini Trans-Blot Cell (Bio-Rad). Membrane was incubated overnight with anti-CD133 (clone W6B3C1) and with peroxidase-conjugated secondary antibody. Actin was used as a housekeeping protein. The peroxidase reaction was obtained by exposure of membrane in the ECL-Plus Western blot detection system solutions (ECL Plus Western blotting Detection Reagents, GE-Healthcare Bio-Sciences, Italia).

### Statistical analysis

Statistical analysis was performed by one-way ANOVA with post-hoc Bonferroni’s multiple comparison sets. Students’t test was performed for statistical comparison between groups (cells and strains) using software InStat Version 3.05 (GraphPad Software, Inc., La Jolla, CA, USA). Statistical significance was set to p < 0.05.

## Supplementary information

Supplementary material

## Data Availability

The data and images generated during the current study are available from the corresponding author on reasonable request.
