# Supplementary material for: Hyaluronic acid inhibition by 4-methylumbelliferone reduces the expression of cancer stem cells markers during hepatocarcinogenesis
Source: Sci Rep. 2019 Mar 11;9:4026. doi: 10.1038/s41598-019-40436-6 (PMC6411988; doi:10.1038/s41598-019-40436-6)
Supplement: Supplementary file 1 — Supplementary material [file 41598_2019_40436_MOESM1_ESM.pdf]

Hyaluronic acid inhibition by 4-methylumbelliferone reduces the expression of cancer stem cells markers during hepatocarcinogenesis

Caecilia H C Sukowati[1,2*], Beatrice Anfuso[1], Esteban Fiore[3], Susan I Ie[4], Alan Raseni[5], Fulvia Vascotto[5], Claudio Avellini[6], Guillermo Mazzolini[3], Claudio Tiribelli[1]

1. Fondazione Italiana Fegato, AREA Science Park Basovizza, SS14 km 163.5, 34149 Trieste, Italy
2. Department of Medicine. University of Udine. Piazzale M. Kolbe 1, 33100 Udine, Italy
3. Gene Therapy Laboratory, Facultad de Ciencias Biomédicas, Universidad Austral, Avenida Presidente Perón 1500, B1629ODT Derqui-Pilar, Buenos Aires, Argentina
4. Laboratory of Hepatitis and Emerging Diseases, Eijkman Institute for Molecular Biology, Jl. Diponegoro 69, 10310 Jakarta, Indonesia
5. Institute for Maternal and Child Health - Institute for Research and Health Care Burlo Garofolo, Via dell'Istria, 65, 34137 Trieste, Italy
6. Department of Medical and Biological Sciences, University Hospital Santa Maria della Misericordia, Piazzale Santa Maria della Misericordia 15, 33100 Udine, Italy

*Correspondence and requests for materials should be addressed to CHCS

caecilia.sukowati@fegato.it

Supplementary Figure

Fig. S1. Unprocessed original scans of Western blot in Fig 5B.

CD133

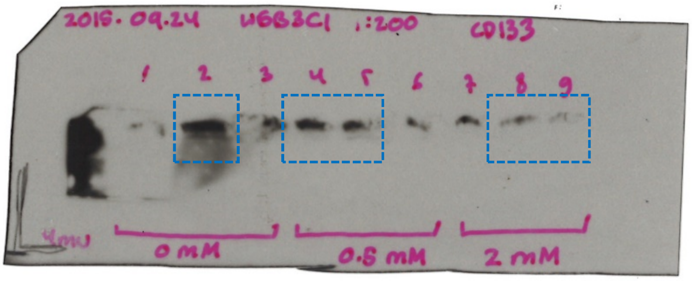

Actin

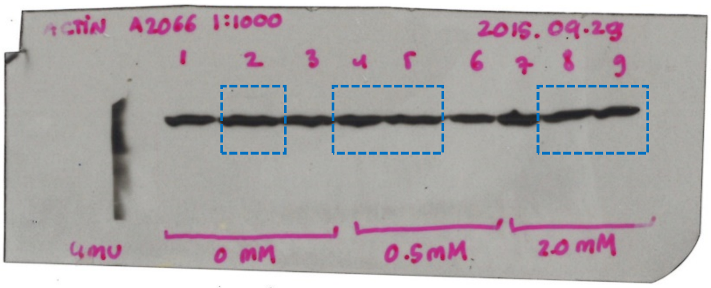

Fig. S2. EMT gene analysis on HCC cell lines treated with 4MU.

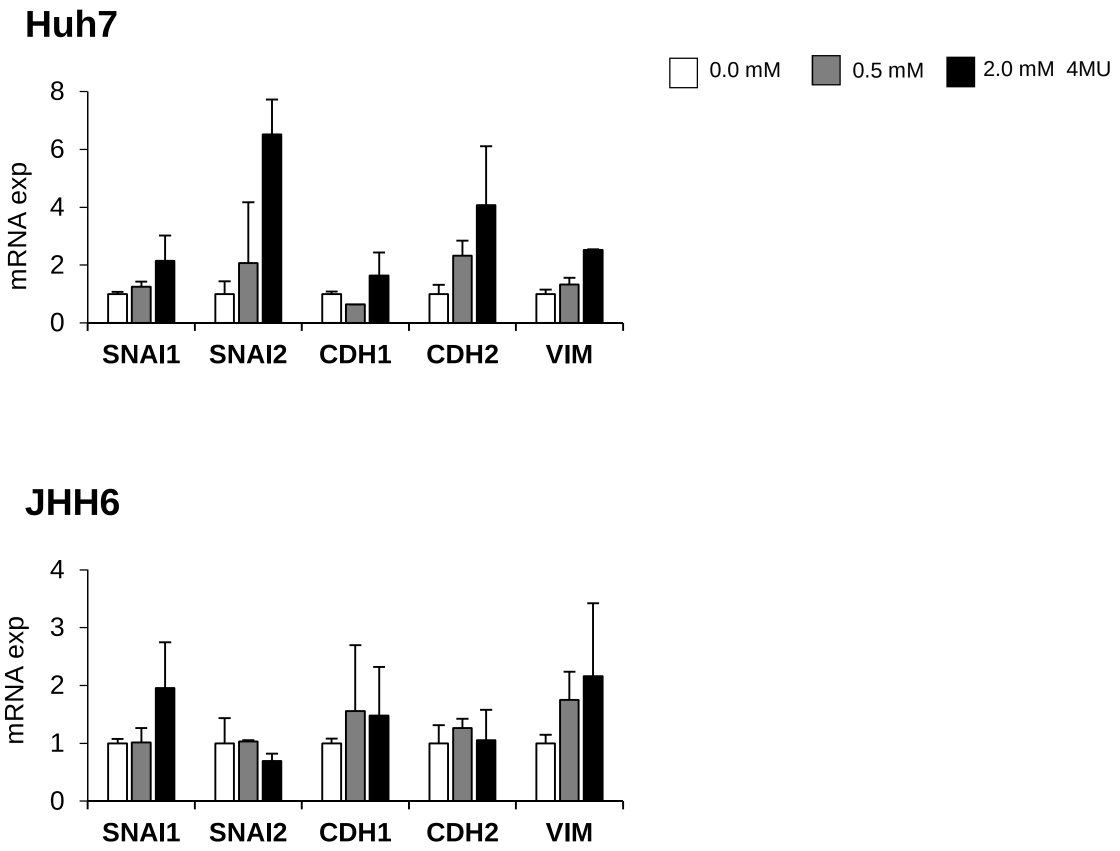

Fig. S3. The staining of HA-coated plate by Alcianblue 8GX showing the positivity of HA.

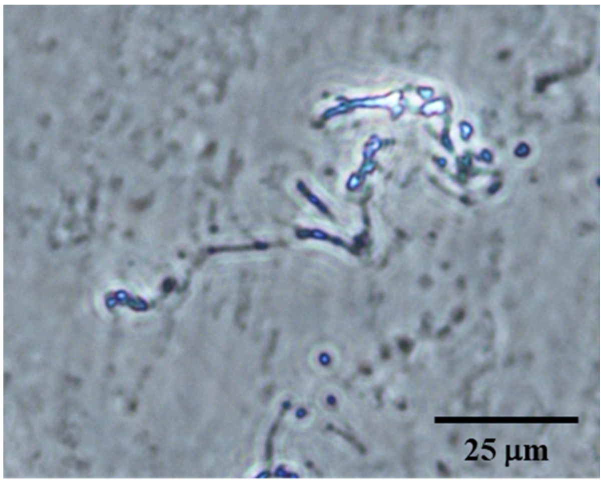

Supplementary Table

Table S1. List of primers used in this study

Gene	Primer F (5' → 3')	Primer R (5' → 3')	Ref
**Human**			
18sr-RNA	TAACCCGTTGAACCCCATT	CCATCCAATCGGTAGTAGCG	[1]
ACTB	CGCCGCCAGCTCACCATG	CACGATGGAGGGGAAGACGG	This study
EpCAM	GAATAATAATCGTCAATGCCAGTG	CGCTCTCATCGCAGTCAG	This study
CD90/THY-1	AGAGACTTGGATGAGGAG	CTGAGAATGCTGGAGATG	This study
CD133/Prominin 1	CATCTGCTCTCTGCTGAC	AACTTAATCCAACTCCAACC	This study
CD44	AGGAAGAAGGATGGATATGGACTC	TTACTCTGCTGCGTTGTCATTG	This study
HAS1	TACAACCAGAAGTTCCTGGG	CTGGAGGTGTACTTGGTAGC	[2]
HAS2	GTGGATTATGTACAGGTTTGTGA	TCCAACCATGGGATCTTCTT	[2]
HAS3	GAGATGTCCAGATCCTCAACAA	CCCACTAATACACTGCACAC	[2]
HYAL1	GATGTCAGTGTCTTCGATGTGGTA	GGGAGCTATAGAAAATTGTCATGTCA	[3]
HYAL2	CTAATGAGGGTTTTGTGAACCAGAATAT	GCAGAATCGAAGCGTGGATAC	[3]
CDH1	GGAACTATGAAAAGTGGGCTTG	AAATTGCCAGGCTCAATGAC	[4]
CDH2	GACGGTTCGCCATCCAGAC	TCGATTGGTTTGACCACGG	[5]
VIM	AACTTCTCAGCATCACGATGAC	TTGTAGGAGTGTCGGTTGTTAAG	This study
BCL2a	GTGTGTGGAGAGCGTCAAC	CGGTTCAGGTACTCAGTCATC	This study

BAX	TCGCCCTTTTCTACTTTG	CCCATGATGGTTCTGATC	This study
PUMA	CCTGTAAGATACTGTATATGC	CCACTGTTCCAATCTGAT	This study
**Mouse**			
Gapdh	CCAGTATGACTCCACTCACG	CTCGCTCCTGGAAGATGGTG	[6]
Actb	AATAAGTGGTTACAGGAAGTC	ATGAAGTATTAAGGCGGAAG	[7]
Has1	TTCCACTGTGTGTCCTGCAT	TGTACCAGGCCTCCAAGAAC	[8]
Has2	GGGACCTGGTGAGACAGAAG	ATGAGGCAGGGTCAAGCATA	[8]
Has3	TCCCCAAGTAGGAGGTGTTG	TTGCACACAGCCAAAGTAGG	[8]
Hyal1	CCGTAATGCCCTACGTCCAGA	GCCTGGCATGATTCCTTGGT	[8]
Hyal2	AGCCGCAACTTTGTCAGTTT	GAGTCCTCGGGTGTATGTGG	[8]
Cd44	CTCCTGAAGAAGACTGTA	CACGGTTGACAATAGTTAT	This study
Cd90	AACTTCACCACCAAGGAT	TTGTCTCTATACACACTGATACT	[8]
Epcam	ATTGTGGTGGTGTCATTAG	TCCTTTATCTCAGCCTTCT	This study
Fsp1	CAGAAGGTGATGAGCAACT	AGGACAGGAAGACACAGTA	This study
Acta2	GGCATCAATCACTTCAAC	TCTGGTCACCTGTATGTA	This study
CD133/Prominin 1	GACATCTCAGTTGATTCCAAGG	CATGGCGCATTCTGCTTCTGC	[9]

References:

1. Schmittgen, T. D. & Zakrajsek, B. A. Effect of experimental treatment on housekeeping gene expression: validation by real-time, quantitative RT-PCR. *J. Biochem. Biophys. Methods* **46**, 69–81 (2000).

2. Twarock, S. *et al.* Synthesis of hyaluronan in oesophageal cancer cells is uncoupled from the prostaglandin-cAMP pathway. *Br. J. Pharmacol.* **157**, 234–243 (2009).
3. Li, L., Asteriou, T., Bernert, B., Heldin, C. H. & Heldin, P. Growth factor regulation of hyaluronan synthesis and degradation in human dermal fibroblasts: importance of hyaluronan for the mitogenic response of PDGF-BB. *Biochem. J.* **404**, 327–336 (2007).
4. Lin, C. W. *et al.* Epithelial cell adhesion molecule regulates tumor initiation and tumorigenesis via activating reprogramming factors and epithelial-mesenchymal transition gene expression in colon cancer. *J. Biol. Chem.* **287**, 39449–39459 (2012).
5. Kroepil, F. *et al.* Down-regulation of CDH1 is associated with expression of SNAI1 in colorectal adenomas. *PloS One* **7**, e46665 (2012).
6. Hayashi, A., Suzuki, H., Itoh, K., Yamamoto, M. & Sugiyama, Y. Transcription factor Nrf2 is required for the constitutive and inducible expression of multidrug resistance-associated protein 1 in mouse embryo fibroblasts. *Biochem. Biophys. Res. Commun.* **310**, 824–829 (2003).
7. Raab, R. M., Bullen, J., Kelleher, J., Mantzoros, C. & Stephanopoulos, G. Regulation of mouse hepatic genes in response to diet induced obesity, insulin resistance and fasting induced weight reduction. *Nutr. Metab.* **2**, 15 (2005).
8. Hunt, L. C. *et al.* Hyaluronan synthesis and myogenesis: a requirement for hyaluronan synthesis during myogenic differentiation independent of pericellular matrix formation. *J. Biol. Chem.* **288**, 13006–13021 (2013).
9. Kemper, K., Tol, M. J. P. M. & Medema, J. P. Mouse tissues express multiple splice variants of prominin-1. *PloS One* **5**, e12325 (2010).
